# Substance Use among Economically Disadvantaged African American Older Adults; Objective and Subjective Socioeconomic Status

**DOI:** 10.3390/ijerph16101826

**Published:** 2019-05-23

**Authors:** Shervin Assari, James Smith, Ritesh Mistry, Mehdi Farokhnia, Mohsen Bazargan

**Affiliations:** 1 Departments of Family Medicine, College of Medicine, Charles R Drew University of Medicine and Science, Los Angeles, CA 90059, USA; jamessmith@cdrewu.edu (J.S.); mohsenbazargan@cdrewu.edu (M.B.); 2 Department of Health Behavior and Health Education, School of Public Health, University of Michigan, Ann Arbor, MI 48109, USA; riteshm@umich.edu; 3 Section on Clinical Psychoneuroendocrinology and Neuropsychopharmacology, National Institute on Alcohol Abuse and Alcoholism and National Institute on Drug Abuse, National Institutes of Health, Bethesda, MD 20892, USA; mehdi.farokhnia@nih.gov; 4 Departments of Family Medicine, University of California, Los Angeles (UCLA), Los Angeles, CA 90095, USA

**Keywords:** African Americans, Blacks, older adults, socioeconomic status, socioeconomic position, educational attainment, financial difficulty, smoking, drinking

## Abstract

*Purpose*. This study investigated the effects of objective and subjective socioeconomic status (SES) indicators on two health behaviors, cigarette smoking and alcohol drinking, among African American older adults. *Methods.* This community-based study recruited 619 economically disadvantaged African American older adults (age ≥ 65 years) residing in South Los Angeles. Structured face-to-face interviews were conducted to collect data. Data on demographic factors (age and gender), subjective SES (financial difficulties), objective SES (educational attainment), living arrangement, marital status, healthcare access (insurance), and health (number of chronic medical conditions, self-rated health, sick days, depression, and chronic pain) and health behaviors (cigarette smoking and alcohol drinking) were collected from participants. Logistic regressions were used to analyze the data. *Results.* High financial difficulties were associated with higher odds of smoking cigarettes and drinking alcohol, independent of covariates. Educational attainment did not correlate with our outcomes. Similar patterns emerged for cigarette smoking and alcohol drinking. *Conclusion*. Subjective SES indicators such as financial difficulties may be more relevant than objective SES indicators such as educational attainment to health risk behaviors such as cigarette smoking and alcohol drinking among African American older adults in economically constrain urban environments. Smoking and drinking may serve as coping mechanisms with financial difficulty, especially among African American older adults. In line with the minorities’ diminished returns (MDR) theory, and probably due to discrimination against racial minorities, educational attainment has a smaller protective effect among economically disadvantaged African American individuals against health risk behaviors.

## 1. Introduction

### 1.1. Background

Smoking is the leading preventable behavioral risk factor of early mortality in the United States [1]. Smoking is associated with anxiety [2], depression [3], low quality of life [4], chronic medical conditions (CMC) [5], and mortality [1]. For example, smoking increases the risk of lung [6], breast, cervical [7], pancreatic [5], gastric [8], and oral [6] cancer. Smoking also increases the risk of hypertension [9], heart disease [10], and stroke [11]. Smoking is particularly consequential in older adults, as it can also increase risk of pneumonia and cognitive decline [12]. Many of these effects may also be seen with alcohol drinking. Drinking alcohol increases risk of cognitive decline [13] and some types of cancer such as renal, liver, and colon [14]. Smoking and drinking are particularly detrimental to older adults, who may be more fragile [15].

However, very little information exists on the epidemiology of these behaviors for low-income African American older adults in urban settings [16,17]. This information is necessary for designing and implementing cessation programs for economically disadvantaged African Americans who use these substances [18,19]. The pattern and predictors of smoking in the African American community may differ from those in other communities. Predatory and targeted advertisement, availability of tobacco outlets, differential patterns of initiation, as well as lower likelihood of access to cessation services in African American majority communities all increase the risk and vulnerability of this population to substance use problems [20,21,22]. As a result of a phenomenon called the telescoping effect, African Americans who engage in substance use are more likely to experience undesired substance use trajectories, which disproportionately increases the risk of undesired consequences [20,23,24,25]. More rapid progression of African Americans relative to that of Whites is shown for a wide range of substances, including opioids [26] and alcohol [26,27]. As a result of this phenomenon, despite a lower prevalence of substance use, the undesired health outcomes associated with substance use are more common in African Americans, which is suggestive of a vulnerability in this population [20,21,22,23,24,25]. That is, smoking cigarette and drinking alcohol are believed to result in more cancer, respiratory, cardiovascular, and liver disease for African Americans than Whites.

Socioeconomic status (SES) is among the strongest social determinants of health [28,29,30,31], with a particularly strong impact on smoking [16] and drinking [32,33]. Scholars and theorists have conducted extensive work on the mechanism by which SES, including educational attainment and financial difficulties, impact health across cohorts [28,29,30,31].

Subjective (i.e., an individual’s evaluation of their own social status and financial and economic power) and objective (i.e., measures that reflect social status such as education, income, and employment) SES both impact health outcomes in African American families, albeit in different ways. Subjective SES, such as financial difficulties, may have large effects on the health outcomes of the African American community that cannot be observed from objective SES indicators alone [34]. Similarly, subjective but not objective SES seems to moderate the effects of discrimination on health for African Americans [34]. At the same time, high levels of objective SES correlate with more but not less discrimination and depression in the African American community [34,35,36,37,38,39]. In this context, high SES may increase psychological vulnerability to racism, which reduces its health-promoting effects for African Americans.

There is a growing body of evidence suggesting that the health implications of objective and subjective SES are unequal across racial and ethnic groups. For example, objective SES (e.g., as seen in measures like educational attainment) has smaller effects for African American individuals [16,40,41,42,43,44] than for Whites, a pattern not unique to African Americans but also observed in Hispanics [32,45] and sexual minorities [46]. As explained by the minorities’ diminished returns (MDR) theory [47], almost every objective SES indicator has shown stronger health effects for Whites than for African Americans [16,42,44,48,49,50,51]. For example, the effects of educational attainment on self-rated health [42], smoking [16], drinking [52], depression [53], and obesity [44] are smaller for African Americans than for Whites. While some of this evidence is based on studies comparing African American and White older adults [52], most of this literature is on younger age groups [16,42,44,48,49,50]. As a result, we know very little about how objective and subjective SES indicators operate for economically disadvantaged African American older adults.

### 1.2. Aims

The current study tested the effects of objective and subjective SES on two health behaviors, cigarette smoking and alcohol drinking, in an economically disadvantaged sample of African American older adults in South Los Angeles. We considered educational attainment and financial difficulties as a proxy for objective and subjective SES, respectively. Built on the MDR theory, we expected a small effect of objective SES and a large effect of subjective SES in our sample. Thus, we expected individuals with high educational attainment and low financial difficulties to report lower levels of smoking and drinking. These hypotheses were based on similar findings that have been observed across health outcomes [20,54]. As suggested by the MDR theory, racism bounds and limits the health gains that are expected to follow objective SES measures such as educational attainment [32,40,41,42,45,47,48,49,50,55]. Conversely, financial difficulties that reflect deep poverty and lack of resources more severely impact health outcomes [56]. This study extends the literature in multiple aspects. First, few comparative studies exist on the effects of objective and subjective SES. Second, few studies have been conducted in low-income African Americans. Finally, most research on substance use is on youth or adults rather than older adults.

## 2. Methods

### 2.1. Design and Setting

A cross-sectional survey was performed in South Los Angeles between 2015 and 2018 to investigate medication-related challenges among economically disadvantaged African American older adults [57]. The survey included a comprehensive evaluation of medications and structured face-to-face interviews. Measures included demographic factors (age and gender), objective SES (educational attainment), subjective SES (financial difficulties), living arrangement, marital status, healthcare access (health insurance), health status (number of CMCs, self-rated health (SRH), sick days, depression, and chronic pain), and health behaviors (cigarette smoking and alcohol drinking).

### 2.2. Participants and Sampling

A convenience sample of economically disadvantaged African American older adults was recruited from multiple predominantly African American housing units and senior centers located in South Los Angeles. Participants were eligible if they were African American/Black, non-institutionalized, and aged 55 years or older. Although the main survey included a total number of 740 African Americans 55 years or older, the current analysis only included participants who were 65 years or older (*n* = 619). Exclusion criteria included enrollment in skilled nursing facilities, enrollment in a clinical trial, and considerable cognitive deficits.

### 2.3. Institutional Review Board (IRB)

The IRB at the Charles R. Drew University of Medicine and Science (CDU University IRB #: 14-12-2450-05) approved the study. All participants signed a written informed consent before being enrolled in the study. Participants received financial incentives.

### 2.4. Study Measures

#### 2.4.1. Independent Variables

*Socioeconomic status (SES):* Educational attainment was measured as self-reported years of schooling and was operationalized as an interval variable (higher objective SES).

*Financial difficulty* or lower subjective SES was measured using three questions in line with Pearlin’s list of chronic financial difficulties experienced by low-SES individuals [58,59], such as not having enough money for essential needs like food, clothes, rent/mortgage, utility bills, etc. Responses were on a 5-point Likert-type scale ranging from ‘never’ to ‘always’. A total score was calculated with a higher score reflecting more financial difficulties. The Cronbach’s alpha for this study was excellent (alpha = 0.923).

#### 2.4.2. Covariates

*Demographic Characteristics.* Age and gender were the demographic covariates in this study. Age was treated as an interval variable. Gender was treated as a dichotomous variable.

*Living Arrangement and Family Type*. Two variables reflected participants’ living arrangement and family type. Participants’ living arrangements were measured using a single item. Participants mentioned whether they are living alone or if there were any other members accompanying them. Living arrangement and loneliness are strong determinants of health outcomes among older adults [60]. Participants’ family type (marital status) was measured as a dichotomous variable, where married families/individuals were coded as 1 and any other situation was coded as 0.

*Health Insurance Status.* Using self-reported data, we asked participants if they had any health insurance. This included Medicare, Medicaid, VA, and any nonfederal health insurance, including private insurance. Using this information, we calculated a dichotomous variable that reflected having or not having health insurance, regardless of type. As only 6 individuals reported no insurance, we did not include this variable in our multiple linear regression models.

*Chronic Medical Conditions (CMCs).* Individuals were asked if a physician had ever told them that they have any of these conditions: Hypertension, diabetes, thyroid disorder, cancer, heart disease, asthma, osteoarthritis, chronic obstructive pulmonary disease, rheumatoid arthritis, gastrointestinal disease, or lipid disorder/hypercholesterolemia. Self-reported assessment has been shown to be a valid measure to collect data on CMCs [61,62], although some bias is to be expected. Our measure was the number of CMCs reported by each individual. Our CMC score ranged from 0 to 11, with a higher score indicating higher number of CMCs.

*Self-Rated Health (SRH).* Participants were asked, “In general, would you say your health is: ’very good,’ ‘good,’ ‘fair,’ ‘bad,’ or ‘very bad.’” Responses were coded as an interval variable ranging from 1 to 5, with a higher score indicating worse SRH. Poor SRH predicts risk of mortality, above and beyond covariates such as socioeconomic status and medical risk factors [63,64,65].

*Sick Days.* A single item was used to assess the frequency of sick days. The item read, “In the past 12 months, how frequently have you been sick?” Responses included never, rarely, sometimes, most of the time, and always, coded as an interval variable ranging from 1 to 5, with a higher score indicating more sick days. Sick days is commonly used as a proxy of health status in the literature [66,67].

*Depression.* This study used the 15-item Geriatric Depression Scale (Short Form) (GDS-SF) to evaluate depression [68,69,70]. Responses were on a “yes” or “no” scale. A sum score was calculated with a potential range between 0 to 15. A higher score was suggestive of the presence of more depressive symptoms. The GDS-SF has excellent reliability and validity. This measure has been extensively used to measure depression among older adults in both community and clinical settings [68,69,70].

*Chronic Pain.* We measured chronic pain by four subscales of the McGill Pain Questionnaire– Short Form 2 (MPQ-SF-2) [71]. During face-to-face interviews, participants responded to 22 pain items that asked about the rate and extent to which they experienced various types of pain in the past week. Each item was on a 11-point numeric rating scale from 0 (none) to 10 (worst possible). The subscales of the MPQ-SF-2 include: (a) Continuity (throbbing, cramping, gnawing, aching, heavy, and tender pain), (b) intermittence (shooting, stabbing, sharp, splitting, electric-shock, and piercing pain), (c) neuropathic nature (hot-burning, cold-freezing, itching, tingling or “pins and needles,” light touch, and numbness pain), and (d) affective domain (tiring-exhausting, sickening, fearful, and punishing-cruel pain). We calculated a total pain score, based on averaging responses to all questions [71,72]. A higher score is indicative of more intense chronic pain.

#### 2.4.3. Outcome Variables

*Health Behaviors.* Participants were asked whether they smoke cigarettes and whether they drink alcohol. The exact questions were: “How would you describe your cigarette smoking habits?” and “Do you drink alcohol?” Response items to the first question was never smoked, previously smoked, and current smoker. The answer to the second questions was yes and no. These two variables were operationalized as dichotomous variables. Our smoking variable was current smoker 1 versus any other condition 0.

### 2.5. Statistical Analysis

We used SPSS 23.0 (SPSS Inc., Chicago, IL, USA) for data analysis. To describe the sample, we reported frequencies (*n*), relative frequencies (%), means, and standard deviations (SD). We used the Pearson correlation test (zero order correlation) to estimate the bivariate correlations between the study variables. These bivariate correlations helped us to rule out multicollinearity between our predictors. We applied logistic regression models with health behavior outcomes (cigarette smoking and alcohol drinking) as the dependent variables, educational attainment and financial difficulties as the independent variables, and demographic factors (age and gender), living arrangement, marital status, and health status (CMC, SRH, sick days, depression, and chronic pain) as covariates. These confounders were determined based on the literature review. For example, living arrangement and marital status may confound the association between SES and health behaviors [47,73]. Similarly, some older adults may decide to change their substance use upon being diagnosed with a chronic medical condition [74]. As only six individuals reported not having any health insurance, we decided not to include this variable in our logistic regression models. We reported odds ratio (OR), standard error (SE), 95% confidence intervals (95% CI), and *p* values.

## 3. Results

### 3.1. Descriptive Statistics

Table 1 describes the study variables in the sample. All participants were 65 years or older and 74.0 years old on average. Participants were mostly females (65.3%), lived alone (59.9%) and nonmarried (85.8%). Almost all participants had some type of health insurance (99%). Of all participants, 15.5% and 30.0% reported cigarette smoking and alcohol drinking, respectively.

### 3.2. Bivariate Analysis

Table 2 shows the results of bivariate correlations between study variables. This table reports Pearson correlation coefficients and *p*-value levels. Educational attainment and financial difficulties were negatively correlated (r = −0.13, *p* < 0.01), suggesting that individuals with higher educational attainment experienced lower levels of financial difficulties. Financial difficulties were positively correlated with both smoking (r = 0.15, *p* < 0.01) and drinking (r = 0.15, *p* < 0.01). Educational attainment was not correlated with smoking (r = −0.02, *p* > 0.05) and drinking (r = 0.05, *p* > 0.05). Smoking and drinking were also positively correlated (r = 0.27, *p* < 0.05), suggesting that African American older adults who smoke cigarettes are more likely to drink alcohol as well.

### 3.3. Multivariable Analysis

Table 3 shows the results of two logistic regression models, one for smoking cigarettes and one for drinking alcohol as the outcome. According to these models, high levels of financial difficulties were associated with higher odds of smoking cigarettes and drinking alcohol, above and beyond all covariates. These models showed that educational attainment was not associated with odds of smoking cigarettes and drinking alcohol, while confounders were controlled.

## 4. Discussion

In our study, financial difficulties but not educational attainment predicted smoking cigarettes and drinking alcohol among economically disadvantaged African American older adults. This suggests that in economically disadvantaged urban areas, educational attainment fails to protect its residents, at least against substance use. That is, more educated African American older adults remain at the same risk of substance use as their less educated counterparts. At the same time, financial need is associated with smoking cigarettes and drinking alcohol among African American older adults.

This study showed that financial difficulties are a risk factor for smoking and drinking among African American older adults. Chronic financial difficulties are consequential for African Americans [75,76,77], particularly for older adults [78,79,80]. Financial difficulties predict future heart disease in the African American community [81]. The detrimental effects of financial difficulty are highest when the population lacks access to buffering resources, such as social support [82]. This is one reason economically disadvantaged African American older adults may be more vulnerable to financial difficulty.

The association between SES and health behaviors such as smoking and drinking are multidimensional and bidirectional [83]. Some people may use substances to cope with stressors such as financial difficulties. Low SES, both objective and subjective, are proxies of life circumstances. Individuals with low educational attainment may have lower knowledge regarding tobacco harm [84]. Individuals with low SES have worse emotion regulation and may use less healthy coping strategies to deal with their stressors [50]. Individuals with lower SES live in closer proximity to outlets and shops that sell tobacco products and alcoholic beverages [85] and have a higher risk of exposure to point-of-sale advertisements, coupons, and discounts [86,87]. In addition, low-SES individuals may be specially targeted by the tobacco industry through predatory marketing, including but not limited to flavoring [87,88]. Finally, low-SES individuals are more likely to live in areas with less restrictive tobacco policies, defined as lower age for tobacco and alcohol purchase, lower cigarette taxes, and no smoke-free laws [89].

Among low-income African American older adults, variation in financial difficulties impacts cigarette smoking and alcohol drinking. Financial difficulties can be a consequence of structural racism, segregation, combined with low SES. A high level of financial difficulty is a risk factor for poor health in the general population [82], for individuals with CMCs [90] and for older adults [91]. A high level of financial difficulty increases the risk of heart disease [81] and complicates the management of diseases like diabetes [90] and cancer [92,93]. Financial difficulties operate as a stressor and increase oxidative stress [94], limiting available choices needed to maintain health [91]. Financial difficulties limit access to and use of health care services [91] and are a risk factor for health-risk behaviors such as poor diet [95]. Financial difficulties also increase the risk of CMC [81], poor SRH [96], depression [77], smoking cigarettes [97], alcohol use [33], and suicide [98], which are barriers to maintaining health.

Our results showed that high education fails to reduce health-risk behaviors of African American older adults who live in low-income urban settings, which are limited in resources and full of stress. At the same time, the unmet financial needs and financial distress of the African American population strongly impact their health behaviors. The health hazard of poverty and unmet financial needs are well described across populations [75,77], including but not limited to African American older adults [78,80]. African Americans with high levels of financial difficulties are at increased risk for heart disease [81], depression [56], and other health problems. African Americans have not fully benefited from education as a resource for achieving financial security. This is in line with the MDR hypothesis.

Supporting our findings on the undesired effects of financial difficulties on health behaviors, multiple studies have documented the effects of financial strain on cigarette smoking [97] and alcohol use [33]. The effects of financial stress go beyond these two behaviors and expand to self-rated health [96], chronic disease [81], depression [77], suicide [98], and mortality [99,100,101]. These effects are repeatedly documented for the general population [102] and for people with chronic disease [90]. Older adults [91] and individuals who have diabetes [90], cancer [99], and heart disease [100] are hit hard by chronic financial difficulties. All these studies collectively provide undeniable evidence that financial difficulties are one of the strongest social determinants of health [96]. Our findings extend this literature to older African Americans in urban areas that are economically disadvantaged.

Extending our results, financial difficulties increase health problems in various ways. It is a stressor and increases oxidative stress [94]. It reflects availability of money, cash, expendable income, and other financial resources. It limits the choices that are needed to maintain health [91] and worsens health-risk behaviors such as cigarette smoking [97] and alcohol use [33]. It reduces access to healthy food [95] and health care when needed [91]. Thus, long-term financial difficulties become a major constraint in addition to what already limits the lives of low-SES African Americans.

Small or no protective effects of educational attainment and other SES resources on health outcomes is predicted by the MDR theory. These patterns might be due to structural racism across institutions [40,41,42,45,48]. These diminished returns for African Americans [16,40,41,42,44], Hispanics [32,45], and sexual minorities [46] stand in contrast to Whites (the socially dominant group), for whom educational attainment does translate to healthier behaviors and, thus, better health [28,29,30,31].

Although we called our measure “subjective” SES, we do not imply that it is unreal or less real. Subjective SES is a result of real financial pressures in the lived experience of low-income African American geriatric populations. Perceived financial difficulty is a real consequence of low SES, and it is important to assess its impact on health behaviors and overall health.

This study extends the existing literature to economically challenged African American older adults who live in low-income resource scarce areas. Most of the previous studies on the effects of SEP on cigarette smoking [97] and alcohol use [33] are among youth and adult rather than older African Americans. Context matters, and SEP resources may not similarly impact all behaviors across all groups, and understanding the nuances regarding group differences in the risk factors may help program planners to more effectively improve public health of subpopulations.

### 4.1. Implications

This study demonstrates that socioeconomic policy is a critical component of health promotion in underserved African American populations. However, not all social determinants of health have equally causative effects on health disparities. Unless structural factors are addressed, we should not rely on educational attainment alone to protect populations in poor urban areas that have limited resources and are full of all types of stressors, including but not limited to hunger, crime, and unsafety. The results of this study highlight the need for policies providing financial support for individuals who encounter financial emergencies. Even when there is educational attainment, a policy that reduces or buffers financial difficulties is needed for many older adults. Older adults often do not have access to other buffers, such as social support, and may be hit harder by financial difficulties [82].

We argue that economic and social policies that provide financial support may reduce risky behaviors like cigarette smoking and alcohol drinking among the general population, but particularly among African American older adults in urban settings. Policies that provide economic resources or social services are important components of efforts tackling health disparities. Deep chronic poverty compounded with the complexity of multiple chronic diseases adds to the challenge. Policies that increase access to financial support are extremely important [101,103,104]. Unfortunately, such policies are not viewed favorably in the US and are often charged with being communistic based on the assumption that people in poverty should simply pull themselves up by their own bootstraps. Real fundamental changed cannot happen before African Americans and other underrepresented groups have political power to shape discourse about social justice, equity, and relevant economic and public policies [101,103,104]. To make matters worse, studies show that early mortality of African Americans results in a lost opportunity to correct the policies that have the potential to address the problem of poverty among African Americans [101,103,104]. The people who need the policy changes the most have far less capacity to influence policy simply because per capita, African Americans have fewer voting years than Whites due to their lower life expectancy.

Moreover, promotion of educational attainment as a means of addressing health inequities will fail to generate lasting results if other social and structural causes of health disparities are left unaddressed [105]. Policy makers should be aware that merely enhancing education may have less of an impact than expected because racial and minority groups gain fewer health benefits from education than Whites. Interventions should make sure that cross-racial variations exist in the effects of SES indicators such as education [28,29,30,31,73]. If we only address SES and fail to help racial and ethnic minorities to mobilize their SES, we may be surprised to discover that our interventions have not had the desired impact. In addition, while enhancement of educational attainment is still needed as it changes life style and increases health through multiple mechanisms [106], increasing access of people to income is also important. Our findings also suggest that policy makers should focus on reducing absolute poverty, probably more urgently than enhancing access to education. We should also be aware that merely altering educational attainment will not be enough to solve the problem of health disparities, given the evidence that objective SES indicators such as education fail to promote health among African Americans [16,40,41,42,43,44].

The proximal experience of financial difficulties was associated with risk behaviors, while a more distal measure like education was not. Our data showed a very weak association between educational attainment and financial difficulties. This suggests to us that there may be a historical lack of access to good-paying jobs and other opportunities for African Americans, regardless of their education, which is where interventions may focus. Although we called our SES measures subjective and objective, this does not suggest that financial difficulties are only in individuals’ minds and not real (objective). In fact, our finding shows that this “subjective SES” may matter more than education. Thus, these “subjective” measures of SES reflect very real and consequential financial difficulties, leading to lower access to money in retirement age. At the same time, educational attainment had a very weak effect on the financial security in this sample of African American older adults.

There is a need to further study why educational attainment had less than expected protective effects on health behaviors in this sample of African American older adults. It is still unknown which social and economic policies can maximize the health benefits that are expected to follow from educational attainment for African American individuals and families. Policies should be implemented that help to eliminate this aspect of MDR of SES across populations and health outcomes [101,103,104]. However, one policy avenue suggested by this study is addressing poverty as a means of addressing health disparities. We are not aware of many previous attempts to reduce the health disparities of economically disadvantaged African Americans through reducing their financial difficulties. Socioeconomic interventions should be designed, implemented, and evaluated in order to tackle behavioral health disparities in African American communities. This also includes attempts to reduce tobacco and alcohol use and misuse. Future research may also focus on dual use, i.e., individuals who both smoke and drink.

### 4.2. Limitations

The study had several limitations. First, the study used a cross-sectional design, limiting causal inferences. In addition, the study used a nonrandom sample, which reduces generalizability of the results. In addition, the study only used self-reports to measure smoking and drinking. For example, the drinking variable does not allow us to examine whether participants are drinking at risky levels for older adults, whether they have experienced consequences associated with drinking, or if they meet criteria for alcohol use disorder. In addition, we used data on having any insurance, regardless of its type. Future studies may include more detailed information about the types of insurance. It would also be useful to collect more detailed data on the employment and salary experiences of the sample. There is a need to measure retirement savings or income that are necessary to pay for basic needs. Finally, the study only included African Americans. The processes studies here may differ for biracial or multiracial African Americans. More research is needed with more detailed information about race and ethnicity.

Financial difficulties may be more relevant to the health behaviors (smoking and drinking) of economically disadvantaged African American older adults than educational attainment. This might be because urban areas may limit how much health gain can follow educational attainment. Although most of our participants were no longer in the labor market, which carries some of the health gains of educational attainment through giving participants good-paying jobs, urban areas limit healthy lifestyles that are expected to follow educational attainment.

## 5. Conclusions

In line with the MDR theory, educational attainment generated a less than expected impact on risk behaviors in economically disadvantaged African American older adults. Future research should study how limited resources in the urban areas, residential segregation, or discrimination against racial minorities reduce health gains of educational attainment. Future research should test which economically disadvantaged African American older adults tend to drink and smoke to cope with stress due to financial difficulties and other types of stress. Such studies may conceptualize gender as a factor that shapes how African American older adults cope with economically adverse conditions.

## Figures and Tables

**Table 1:** Descriptive statistics (*n* = 619).

Characteristics			
	Mean	SD	Range
Age (Years)	74.0	7.0	65.0–96.0
Educational Attainment (Years)	12.7	2.4	1.0–16.0
Financial Strain	8.2	4.9	5.0–25.0
Chronic Medical Conditions (CMC)	3.8	1.9	0.0–10.0
Self-Rated Health (SRH)	3.1	1.0	1.0–5.0
Sick Days	2.4	1.0	1.0–5.0
Depressive Symptoms	2.1	2.4	0.0–13.0
Chronic Pain	1.8	2.1	0.0–9.7
	***n***	*%*	
Gender			
Women	404	65.3	-
Men	215	34.7	-
Family Type			
Non-Married	531	85.8	-
Married	88	14.2	-
Living Arrangement [Living Alone]			
No	248	40.1	-
Yes	371	59.9	-
Any Health Insurance			
No	6	1.0	-
Yes	613	99.0	-
Cigarette Smoking (Current)			
No	523	84.5	-
Yes	96	15.5	-
Alcohol Drinking			
No	433	70.0	-
Yes	186	30.0	-

**Table 2:** Bivariate correlations.

	1	2	3	4	5	6	7	8	9	10	11	12	13	14
1 Gender	1	0.07	0.14 **	−0.02	−0.12 **	0.08 *	−0.04	0.10 *	0.05	−0.02	−0.02	0.08	−0.23 **	−0.08 *
2 Age (Years)		1	−0.20 **	−0.10 *	−0.05	0.10 *	−0.01	0.02	−0.16 **	0.11 **	−0.10 *	−0.12 **	−0.28 **	−0.16 **
3 Objective SES (Educational Attainment; Years)			1	−0.13 **	0.06	−0.03	0.08	−0.09 *	−0.03	0.00	−0.07	−0.03	−0.02	0.05
4 Subjective SES (Financial Difficulties)				1	−0.09 *	0.15 **	0.01	0.22 **	0.18 **	−0.13 **	0.31 **	0.29 **	0.15 **	0.15 **
5 Married					1	−0.41 **	−0.01	−0.02	−0.09 *	0.00	−0.05	−0.07	0.04	−0.07
6 Living Alone						1	−0.01	0.07	0.07	−0.04	0.14 **	0.14 **	−0.03	0.07
7 Any Health Insurance							1	0.05	−0.01	−0.01	0.03	0.01	−0.00	−0.01
8 Chronic Medical Conditions								1	0.27 **	−0.27 **	0.32 **	0.44 **	0.02	−0.02
9 Self−Rated Health									1	−0.23 **	0.30 **	0.37 **	0.17 **	0.07
10 Sick Days										1	−0.27 **	−0.33 **	−0.07	0.03
11 Depressive Symptoms											1	0.41 **	0.18 **	0.07
12 Pain Intensity												1	0.08 *	0.12 **
13 Smoking Cigarettes													1	0.27 **
14 Drinking Alcohol														1

* *p* < 0.05, ** *p* < 0.01.

**Table 3:** Summary of two multivariable logistic regression models with smoking cigarettes and drinking alcohol as outcomes.

	OR	95% CI	Wald(df)	*p*	OR	95% CI	Wald(df)	*p*
	Smoking Cigarette				Drinking Alcohol			
Educational Attainment (Years)	0.98	0.88–1.09	0.11(1)	0.740	1.07	0.98–1.17	2.23(1)	0.135
Financial difficulties	1.05	1.01–1.10	4.82(1)	0.028	1.05	1.01–1.09	7.25(1)	0.007
Gender (Female)	0.28	0.17–0.46	24.31(1)	<0.001	0.64	0.44–0.94	5.07(1)	0.024
Age (Years)	0.87	0.83–0.91	31.99(1)	<0.001	0.96	0.93–0.99	8.84(1)	0.003
Marital (Married)	1.30	0.61–2.76	0.47(1)	0.494	0.71	0.39–1.31	1.20(1)	0.274
Living Arrangement (living alone)	0.88	0.49–1.57	0.18(1)	0.669	1.21	0.80–1.84	0.83(1)	0.363
Chronic medical conditions (CMC)	0.94	0.81–1.10	0.59(1)	0.443	0.91	0.81–1.02	2.58(1)	0.108
Self-rated health (SRH)	1.49	1.13–1.95	8.14(1)	0.004	1.06	0.86–1.29	0.27(1)	0.601
Sick days	1.02	0.79–1.32	0.02(1)	0.876	1.22	1.01–1.49	4.17(1)	0.041
Depression	1.14	1.03–1.26	6.33(1)	0.012	1.01	0.92–1.10	0.02(1)	0.884
Chronic Pain	0.97	0.85–1.11	0.22(1)	0.640	1.14	1.03–1.27	6.60(1)	0.010
